# Treatment of a Locally Invasive Cutaneous Fusarium Species Infection With Voriconazole and Liposomal Amphotericin B in a Patient With Relapsed Acute Myeloid Leukemia

**Published:** 2017-07-01

**Authors:** Sara Anne Meyer, Bruce M. Jones

**Affiliations:** South University School of Pharmacy, Savannah, Georgia, and St. Joseph’s/Candler Health System, Savannah, Georgia

## ABSTRACT

**CASE STUDY**

In February 2011, a 69-year-old, 1.91 m² Caucasian male was diagnosed with acute myeloid leukemia (AML). Immediately prior to the diagnosis of AML, the patient was otherwise healthy until he developed multiple infections, including what was reported as combination viral and bacterial upper respiratory infections. The patient also developed a dental abscess after food became lodged between a molar and gum, which was unable to be drained; therefore, the patient was placed on antibiotics, and an internist ordered a complete blood cell count (CBC). The CBC revealed leukocytosis with 24,500 white blood cells (WBC)/mm³, hemoglobin (Hgb) 10.8 g/dL, and a platelet count of 17,000/mm³. The patient said he experienced excessive bleeding from his gums and expressed exudative material from the abscess. 

When diagnosed, the patient had 50% to 60% monoblasts/promonocyte with 30% monoblasts in peripheral blood. The monoblasts were positive for CD33, dim CD13, dim CD64, dim CD14, CD11c, and CD11b. On immunophenotypic analysis, the percentage of abnormal cells was 60% to 65%. CD34, CD117, and human leukocyte antigen (HLA)-DR were not expressed. The T cells showed no pan T-cell antigenic deletion or diminution and no CD4/CD8 subset restriction. FMS-like tyrosine kinase 3 (FLT3) and nucleophosmin (NPM1) were not mutated.

The patient underwent induction chemotherapy after the initial diagnosis consisting of "7+3" with cytosine arabinoside at 100 mg/m² continuous intravenous infusion for 7 days and idarubicin at 12 mg/m² intravenously (IV) on days 1 through 3. The patient’s induction regimen at that time was and is currently widely accepted as the current standard of care. The patient tolerated the induction therapy well and had no evidence of tumor-lysis syndrome. A bone marrow aspiration was performed on day 14, which is the standard of care for monitoring after induction therapy. The bone marrow aspiration showed virtual aplasia, and flow cytometry did not show blasts. The patient received transfusion support as needed. The patient then underwent four cycles of high-dose cytosine arabinoside consolidation and tolerated the treatment well. After induction and consolidation therapies, the bone marrow extraction was negative for residual or recurrent leukemia.

**Patient Presents With Symptoms**

In October 2014, approximately 3 years following initial diagnosis and remission, the patient presented to his oncologist with a low-grade fever and a 2.3-kg weight loss within the past week attributed to decreased oral intake, oral pain, and edema, which resulted in a weight of 99.3 kg. The patient saw his primary care physician the previous day, and a CBC was obtained; it revealed remarkable leukocytosis with 121,000 WBC/mm³, hemoglobin of 4.8 mg/dL, and a platelet count of 15,000/mm³. Six weeks prior to presentation, the patient had traveled to Missouri and developed fatigue and intermittent chest pain, particularly on exertion, with exertional dyspnea and palpitations 3 weeks later. The patient was admitted to the hospital for transfusion, bone marrow aspiration with core biopsy, and to repeat induction chemotherapy. 

On day 9 of admission, the patient developed neutropenic fever without an obvious source. The CBC revealed 400 WBC/mm³ with an absolute neutrophil count (ANC) of 88/mm³ and a maximum temperature of 38.7°C. The patient believed he suffered a spider wound while he was visiting in Missouri. The area was initially red, with a small fluid-filled blister and was healing on his right arm but remained tender. An infectious diseases physician was consulted, and the patient was subsequently initiated on broad-spectrum antimicrobial therapy consisting of intravenous cefepime and vancomycin, as well as oral (PO) fluconazole at 200 mg daily. Blood cultures, urine cultures, procalcitonin, histoplasmosis antigen, and *Aspergillus* Galactomannan antigen were collected. All serology testing performed was negative.

On day 15 of admission, the right arm cellulitis was largely unchanged, with continued intermittent fevers. Cefepime therapy was continued, but other pharmacologic therapy was adjusted to add high-dose oral ciprofloxacin, intravenous vancomycin was changed to intravenous daptomycin, and oral fluconazole was changed to intravenous voriconazole at 6 mg/kg every 12 hours. All cultures and antigen tests continued to yield negative results; however, fever in the setting of profound neutropenia continued. Due to a concern for an atypical infection, a punch biopsy for diagnosis was obtained on day 16 of admission, and the patient underwent irrigation and debridement of the right arm.

**Confirmation of *Fusarium* Infection**

Cultures collected from irrigation and debridement of the arm confirmed *Fusarium* species infection on day 21 of admission. The patient underwent wide-margin surgical resection of the locally invasive lesion, and there was no evidence of any hematogeneous spread. Due to concern for resistance to azole therapy, intravenous liposomal amphotericin B was initiated at 5 mg/kg every 24 hours on day 21 of admission, and voriconazole was then discontinued, along with daptomycin.

A right cellulitis leg wound appeared and was noted on day 23 of admission. The area was not improving, which raised concern for dissemination of *Fusarium* infection. Empiric gram-positive organism coverage with intravenous vancomycin was also added due to the worsening right leg cellulitis. Oral voriconazole was also resumed in addition to liposomal amphotericin B at a dosage of 300 mg every 12 hours for 2 days. The dosage of voriconazole was decreased to 200 mg every 12 hours for 5 days and subsequently increased back to 300 mg every 12 hours for the duration of the admission. Liposomal amphotericin B therapy was continued for 21 days total in combination with voriconazole.

On day 24 of admission, the patient developed a nonproductive cough and bilateral pulmonary infiltrates with spiculated masses seen on a noncontrast chest computed tomography (CT). This raised concerns for atypical fungal pneumonia. Due to the physician’s concern for *Nocardia* species, cefepime therapy was changed to intravenous imipenem/cilastatin, which was continued for 14 days. The patient had persistent neutropenia and developed worsening renal function. After 2 days of broadening antimicrobial therapy, the cough improved slightly.

The patient’s cough and chest x-ray displayed improvement on day 27 of admission and therefore revealed an alternate source of infection than *Fusarium* species. After 3 days of vancomycin therapy, there was no *Staphylococcus aureus* isolated, and vancomycin therapy was changed to amoxicillin/clavulanic acid. The patient also received recombinant granulocyte macrophage colony-stimulating factor support with sargramostim at 500 mg, equivalent to approximately 218 mg/m²/day, IV infusion every 24 hours for 2 weeks, as the product is labeled for AML following induction chemotherapy. The route was subsequently changed to sargramostim subcutaneous (SC) at 500 mg every 24 hours for 4 days.

The patient’s condition began improving significantly with his absolute neutrophil count ≥ 1,000 (1,164/mm³) on admission day 40. He was then transferred to the physical rehabilitation unit due to significant deconditioning during the 49-day inpatient admission. The patient received physical rehabilitation for 7 days and was then discharged home on amoxicillin/clavulanic acid for 7 additional days for the leg cellulitis and treatment with oral voriconazole at 300 mg every 12 hours for at least 1 year. The duration of voriconazole following fusariosis is not clearly defined in the published literature, but it was determined the patient should continue the agent for an extended period secondary to the severity of the infection and severe immunosuppression. The patient continued to follow up with the oncology, surgical, and infectious diseases specialists after discharge (see Table at right).

**Table:**
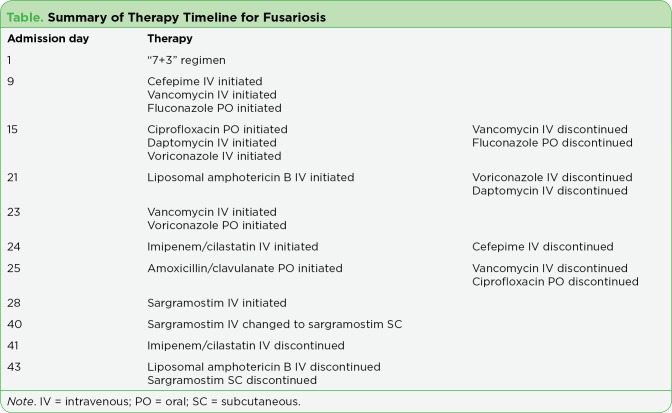
Summary of Therapy Timeline for Fusariosis

## ARTICLE

The *Fusarium* genus, which causes various human diseases, including mycotoxicosis and locally invasive or disseminated infections, is a saprophyte commonly found in soil. Influenced by host immune status, the *Fusarium* fungi may lead to disseminated, life-threatening, disease. In immunocompromised patients, overall mortality associated with disseminated *Fusarium* species ranges from 50% to 80%. Severe immunosuppression, particularly with hematologic malignancies, along with colonization and tissue damage, neutropenia, lymphopenia, graft-vs.-host disease, and corticosteroid therapy are significant risk factors for developing disseminated fusariosis (Dignani & Anaissie, 2004).

Infection with *Fusarium* species in the hematologic cancer patient population is a common mold infection, second only in frequency of infection with *Aspergillus* species. The *Fusarium* species is contracted by the airborne route or the breakdown of skin barriers, which will be the focus of this case report. Fusariosis may respond to novel therapies but presents similarly to aspergillosis, and patients may therefore be treated with conventional amphotericin B deoxycholate monotherapy, which does not have strong established activity against fusariosis. Novel therapies must be explored in these cases, as *Fusarium* may be obstinate to standard antifungal therapies (Boutati & Anaissie, 1997).

A high suspicion for locally invasive cutaneous fusariosis should be raised when severely immunocompromised patients present with extremity cellulitis or subcutaneous lesions are present. The skin is an extremely important source for diagnosing fusariosis. *Fusarium* spp. may appear similar to *Aspergillus* spp. as members of the hyalohyphomycosis family, and both invade blood vessels, which lead to thrombosis and tissue infarction. Both types of fungi also appear in the tissue as acute branching septate hyphae. To definitively diagnose fusariosis, immunohistologic staining must be utilized with polyclonal fluorescent antibody reagents that distinguish *Aspergillus* spp. from *Fusarium* spp. To distinguish the two fungi from each other in an alternative manner, in situ hybridization may be utilized in tissue secretions with a 100% positive predictive value (Dignani & Anaissie, 2004). 

As soon as localized fusariosis is diagnosed, surgical debridement must be performed promptly to prevent seeding of the *Fusarium* species into the bloodstream and thus preventing potentially fatal disseminated fusariosis. The optimal systemic pharmacologic therapy continues to remain unclear. Voriconazole, itraconazole, and the polyenes, more specifically high-dose conventional amphotericin B or liposomal amphotericin B, have been associated with some success in the treatment of fusariosis (Dignani & Anaissie, 2004). The activity of azoles against *Fusarium* species is variable, and the species may sometimes be resistant to amphotericin B (Kanafani & Perfect, 2008). The only agent with an indication for treating refractory fusariosis is voriconazole. Due to increasingly fatal infections with *Fusarium* species, the infection must be detected early and treated aggressively, as *Fusarium* species are considered one of the most drug-resistant fungi (Dignani & Anaissie, 2004). 

The development of skin lesions is one of the most frequently discovered aspects of infections caused by *Fusarium* species and is often the only source of diagnostic material. Due to this reason, it is of utmost importance to distinguish the pathologic features of skin infection involvement in fusariosis (Nucci & Anaissie, 2002). In a study performed by Nucci and Anaissie (2002), patients who were diagnosed with cutaneous fusariosis with persistent neutropenia had a mortality rate of 100%. The results supported a recommendation that local debridement along with biopsy and culture of areas with skin breakdown be performed as soon as possible. 

## CASE DISCUSSION

Previous case reports and studies have not shown a significant role for combination voriconazole and liposomal amphotericin B in vivo, particularly for locally invasive *Fusarium* species infections, although there have been reports of successful treatments in combination with surgery and granulocyte infusions for disseminated disease (Córdoba et al., 2008; Durand-Joly et al., 2003; Ortoneda, Capilla, Pastor, Pujol, & Guarro, 2004). The immediate wide-section surgical excision and debridement, along with the addition of granulocyte infusions, certainly contributed to the successful treatment of this patient.

The description of this case study displays a potential role for synergistic voriconazole with liposomal amphotericin B for the treatment of locally invasive *Fusarium* species infections. The patient in this case study did not improve with antifungal monotherapy. The importance of early detection and aggressive debridement and antifungal treatment was displayed in this case study, which prevented a disseminated infection. Although this case report was limited by the unavailability of final fungi identification from the facility laboratory, a preliminary result of *Fusarium* species indeed warrants immediate surgical excision and potentially combination antifungal therapies vs. monotherapy. 

Awareness and early detection of this opportunistic infection in hematologic cancer patients are of paramount importance to prevent dissemination of the infection and decrease the significant mortality associated with disseminated *Fusarium* infections. The patient in this case study was fortunate with early identification and immediate wide-margin excision of the infection, granulocyte infusions, and aggressive combination antifungal therapy. 

Prolonged neutropenia is associated with disseminated fusariosis and substantial mortality (Nucci & Anaissie, 2002), and colony-stimulating factor support should be considered in patients with fusariosis. The patient in this case study was admitted on two occasions for consolidation therapy for AML after being treated for the locally invasive fusariosis. During the consolidation phases, the patient was still awaiting a match for bone marrow transplant. For consolidation therapy, the patient underwent high-dose cytarabine at 1.5 g/m² every 12 hours on days 1, 3, and 5 for two cycles. The patient also underwent a skin graft on the right forearm, where the locally invasive *Fusarium* infection was excised. During the two consolidation therapies, the patient experienced no further complications or evidence of recurrent or disseminated *Fusarium* species.
